# The Relationship Between Late Morbidity and Dose–Volume Parameter of Rectum in Combined Intracavitary/Interstitial Cervix Cancer Brachytherapy: A Mono-Institutional Experience

**DOI:** 10.3389/fonc.2021.693864

**Published:** 2021-07-23

**Authors:** Ning Zhang, Ying Liu, Dongmei Han, Xin Guo, Zhuang Mao, Wei Yang, Guanghui Cheng

**Affiliations:** Department of Radiation Oncology, China-Japan Union Hospital of Jilin University, Changchun, China

**Keywords:** dose–effect relationship, late rectal complication, cervical cancer, brachytherapy, intracavitary/interstitial brachytherapy

## Abstract

**Purpose:**

To establish a dose volume–effect relationship for predicting late rectal complication (LRC) in locally advanced cervical cancer patients treated with external beam radiotherapy (EBRT) followed by combined intracavitary/interstitial brachytherapy (IC/IS-BT).

**Materials and Methods:**

A retrospective analysis was performed in 110 patients with locally advanced cervical cancer who underwent definitive radiotherapy combined with IC/IS-BT from July 2010 to September 2018. We report the 90% of the target volume receiving the minimum dose for high risk clinical target volume (HR-CTV D_90_) and intermediate risk clinical target volume (IR-CTV D_90_), and the minimum doses to the most exposed 0.1, 1, and 2 cm³ ${\text{D}}_{0.1\text{c}{\text{m}}^{3}},{\text{D}}_{1\text{c}{\text{m}}^{3}},{\text{D}}_{2\text{c}{\text{m}}^{3}}$ doses at the International Commission on Radiation Units and Measurements (D_ICRU_) for organs at risk (OARs). The total dose of EBRT plus brachytherapy was transformed to the biologically equivalent dose in 2 Gy fractions (EQD2) with α/β value of 10 Gy for target, 3 Gy for organs at risk using the linear quadratic model. The morbidity was scored according to the Radiation Therapy Oncology Group (RTOG) criteria. The Probit model was used to establish a prediction model on rectum between the organs at risk for dose and LRC. The receiver operating characteristic (ROC) curve was used to evaluate the predictive value of dose volume parameters for LRC.

**Results:**

The median follow-up time was 72.3 months. The mean ( ± standard deviation) ${\text{D}}_{2\text{c}{\text{m}}^{3}},{\text{D}}_{1\text{c}{\text{m}}^{3}},{\text{D}}_{0.1\text{c}{\text{m}}^{3}}$, and D_ICRU_ values of rectum were 64.72 ± 7.47 Gy_EQD2_, 70.18 ± 5.92 Gy_EQD2_, 79.32 ± 7.86 Gy_EQD2_, and 67.22 ± 7.87 Gy_EQD2_, respectively. The Probit model showed significant relationships between ${\text{D}}_{1\text{c}{\text{m}}^{3}}\text{ or }{\text{D}}_{0.1\text{c}{\text{m}}^{3}}$, and the probability of grade1–4, grade 2–4 rectal events at 1 year, and between ${\text{D}}_{1\text{c}{\text{m}}^{3}}$and the probability of grade2–4 rectal events at 3 and 5 years. The dose values for 10% complication rates (ED10) of ${\text{D}}_{1\text{c}{\text{m}}^{3}}$were 74.18 (70.42–76.71) Gy_EQD2_, 67.80 (59.91, 71.08) Gy_EQD2_, 66.37 (52.00, 70.27) Gy_EQD2_ for grade 2–4 with rectal morbidity at 1, 3, and 5 years, respectively.

**Conclusion:**

Our study proved that ${\text{D}}_{1\text{c}{\text{m}}^{3}}\text{ and}{\text{D}}_{0.1\text{c}{\text{m}}^{3}}$were considered as useful dosimetric parameters for predicting the risk of grade1–4 and grade2–4 LRC at 1-year, and ${\text{D}}_{1\text{c}{\text{m}}^{3}}$might be an indicator for predicting grade2-4 LRC at 3/5years. The patients with rectal ${\text{D}}_{1\text{c}{\text{m}}^{3}}$>66.37–74.18 Gy_EQD2_ should be closely observed for grade2–4 LRC.

## Introduction

Brachytherapy is a crucial component of radical radiotherapy of locally advanced cervical cancer (1), and it mainly includes intracavitary brachytherapy (ICBT), interstitial brachytherapy (ISBT), and hybrid intracavitary/interstitial brachytherapy (IC/IS-BT). The IC/IS-BT approach allows placement of interstitial needles for better coverage of large or asymmetric tumors, delivering a high dose to the target region while sparing the OARs. During brachytherapy, the rectum is one of the most important parts for dose constraints for OARs. It may be helpful to find out a sensitive indicator in dosimetric parameters for rectal assessment of dose to avoid late rectal complication (LRC). Several studies have reported a statistically significant correlation between ${\text{D}}_{2\text{c}{\text{m}}^{3}}$ and occurrence of LRC in cervical cancer patients who had brachytherapy (2, 3). Meanwhile, several other studies found other dosimetric parameters, such as ${\text{D}}_{1\text{c}{\text{m}}^{3}}$ or D_ICRU_, also exhibited a statistically significant relationship (4, 5). However, few studies have compared the differences in rectal dosimetric parameters, while very few lacked a more reliable prediction model. Hence, in the present study, the role of three-dimensional dosimetric parameters ${\text{D}}_{2\text{c}{\text{m}}^{3}},{\text{D}}_{1\text{c}{\text{m}}^{3}},{\text{D}}_{0.1\text{c}{\text{m}}^{3}}$, and two-dimensional dosimetric parameter D_ICRU_ in the prediction of LRC were analyzed and compared, and relevant models for locally advanced cervical cancer treatment with curative radiotherapy including IC/IS-BT were established.

## Materials and Methods

### Patients

A total of 110 patients with locally advanced cervical cancer who visited our hospital and underwent definitive chemoradiotherapy combined with IC/IS-BT were enrolled from July 2010 to September 2018. Most of the patients (94.5%) had squamous cell carcinoma, and the remaining patients had adenocarcinoma (3.64%) or other carcinomas (1.86%). The age of the patients ranged between 23 and 84 years; the mean age at initial treatment was 53.98 ± 10.97. According to 2009 FIGO stage, 6 patients were in IB2, 20 were in IIA2, 59 were in IIB, 8 were in IIIA, 14 were in IIIB, and 3 were in IVA.

### Treatment Procedure

All patients completed external beam radiotherapy with 43.2–55.8 Gy for 25–31 fractions of 1.8–2.0 Gy with or without platinum-based chemotherapy followed by IC/IS-BT with 14–28Gy for 2–4 fractions, 7 Gy one fraction. Eighty-eight patients underwent EBRT technique with IMRT, and the remaining 22 patients completed with 3D-CRT technique. In addition, patient and tumor characteristics and radiotherapy regimen are shown in **Supplementary Table 1**.

IC/IS-BT was based on MRI/CT, and the MRI was performed separately before external beam radiotherapy (EBRT) and brachytherapy treatment. The applicator insertion was completed under general anesthesia in an operating room. The applicators, including the Utrecht applicator combined with interstitial technique, Vienna applicator for combined IC/IS-BT, and the multi-channel vaginal applicator, were used in most of the patients. In patients with advanced stage IIIB, parametrial implants could be added in addition to a standard applicator.

### Data Collection and Evaluation Criteria

According to GYN GEC ESTRO recommendations (6) and Dimopoulos et al. (7) the gross tumor volume (GTV), two clinical target volumes (CTVs, high risk CTVs, intermediate risk CTVs), and outer walls of OARs were delineated based on MRI or CT images. The OARs included the rectum, bladder, sigmoid colon, and small intestine. The target volume and OARs were contoured by one gynecologic doctor then evaluated by two senior gynecologic oncologists in this research, and the doctors took extra care with the rectal contouring. Target volume parameters D_90_ and D_98_ of HR-CTV, IR-CTV were tabulated. ${\text{D}}_{2\text{c}{\text{m}}^{3}},{\text{D}}_{1\text{c}{\text{m}}^{3}},{\text{D}}_{0.1\text{c}{\text{m}}^{3}}$, and D_ICRU_ were tabulated for OARs.

For dose evaluation, the dose calculation was performed using the linear quadratic model with *α*/*β* = 10 Gy for the tumor target, and *α*/*β* = 3 Gy for the OARs, and then converted into the equivalent dose in 2 Gy fractions (EQD2). Total dose evaluation for the CTVs and OARs was the total EQD2 value of the dose from BT accumulated with the dose of EBRT. The goal of combined EBRT and BT was to achieve a total dose ≥85 Gy of HR-CTV D_90_ and a constraint of 85 Gy for the bladder and 70 Gy for the rectum, sigmoid, and bowel.

### Follow-Up

After treatment, all patients were followed up once every 3 months in the first 2 years and once every half a year thereafter. Follow-up evaluation consisted of local recurrence, distant metastasis, survival, *etc.* Late side effects were graded according to the toxicity criteria of the Radiation Therapy Oncology Group (RTOG) and the European Organization for Research and Treatment of Cancer (EORTC) (8). CT/MRI of the pelvis or PET scans were performed once or twice a year according to the specific conditions of these patients.

### Statistical Analysis

The continuous variables were presented as means ± standard deviation (±S) (), and the categorical variables were presented as counts or percentages (%). A Probit regression model was used to investigate the relationships between ${\text{D}}_{2\text{c}{\text{m}}^{3}},{\text{D}}_{1\text{c}{\text{m}}^{3}},{\text{D}}_{0.1\text{c}{\text{m}}^{3}}$, D_ICRU_ and late side effects of rectum, and the receiver operating characteristic (ROC) curve was used to evaluate and compare the predictive values. All statistical analysis was two-sided and performed using SPSS 22.0 software (IBM, Armonk, NY, USA) and Stata 15.0 software (StataCorp LLC, Texas, USA). The EpiData 3.10 software (EpiData Association, Odense M, Denmark) was used for data entry and database establishment.

## Results

### Patient Characteristics

Brachytherapy was conducted for a total of 438 times. The average number of needles per fraction was 3.78 (range 1–8), and the average depth was 3.12 cm. The ${\text{D}}_{2\text{c}{\text{m}}^{3}},{\text{D}}_{1\text{c}{\text{m}}^{3}},{\text{D}}_{0.1\text{c}{\text{m}}^{3}}$, and D_ICRU_ for rectum, HR-CTV D_90_, and IR-CTV D_90_ are presented in **Table 1**. In addition, 17 cases of the 110 patients with cervical cancer did not receive chemotherapy, 21 cases received neoadjuvant chemotherapy before radiotherapy, 34 cases received concurrent chemotherapy, 1 case received adjuvant chemotherapy after radiotherapy, 25 cases received neoadjuvant chemotherapy combined with concurrent chemotherapy, 9 cases received concurrent chemotherapy combined with adjuvant chemotherapy, and 3 cases received neoadjuvant chemotherapy combined with concurrent chemotherapy and adjuvant chemotherapy. All chemotherapy regimens were platinum based. Other characteristic information and clinical outcomes have been previously published in scholarly journals (9).

**Table 1:** DVH parameters for HR-CTV and OARs.

DVH parameters	$\bar{\text{x}}$± S(Gy_EQD2_)
HR-CTV D_90_	91.28 ± 8.63
IR-CTV D_90_	68.47 ± 3.79
$\text{Bladder }{\text{D}}_{2\text{c}{\text{m}}^{3}}$	77.20 ± 7.04
$\text{Bladder }{\text{D}}_{1\text{c}{\text{m}}^{3}}$	82.36 ± 6.42
$\text{Bladder }{\text{D}}_{0.1\text{c}{\text{m}}^{3}}$	93.95 ± 9.29
Bladder D_ICRU_	76.72 ± 11.48
$\text{Rectum }{\text{D}}_{2\text{c}{\text{m}}^{3}}$	64.72 ± 7.47
$\text{Rectum }{\text{D}}_{1\text{c}{\text{m}}^{3}}$	70.18 ± 5.92
$\text{Rectum }{\text{D}}_{0.1\text{c}{\text{m}}^{3}}$	79.32 ± 7.86
Rectum D_ICRU_	67.22 ± 7.87

DVH, dose volume histogram; HR-CTV, high risk clinical target volume; IR-CTV, intermediate risk clinical target volume; D_90,_ the minimum dose delivered to 90% of the target volume; ${\text{D}}_{2\text{c}{\text{m}}^{3}},{\text{D}}_{1\text{c}{\text{m}}^{3}},{\text{D}}_{0.1\text{c}{\text{m}}^{3}}$, *minimal dose to the maximally exposed 2 cm³, 1 cm³, 0.1 cm³ of organs at risk, respectively.* D_ICRU_, *dose delivered to the International Commission for Radiation Units and Measurements (ICRU) point*.

### Tumor Response

The criteria for evaluation of posttreatment efficacy were based on the revision of Response Evaluation Criteria for Solid Tumors published in 2009 (RECSIT 1.1). The treatment efficacy of those 110 patients was assessed after radiotherapy, in which 72 (65.45%) cases achieved complete response (CR), 36 (32.73%) cases achieved partial response (PR), 1 (0.91%) case achieved progressive disease (PD), and 1 (0.91%) case achieved stable disease (SD).

### Incidence of Late Rectum Morbidity

**Figure 1:**
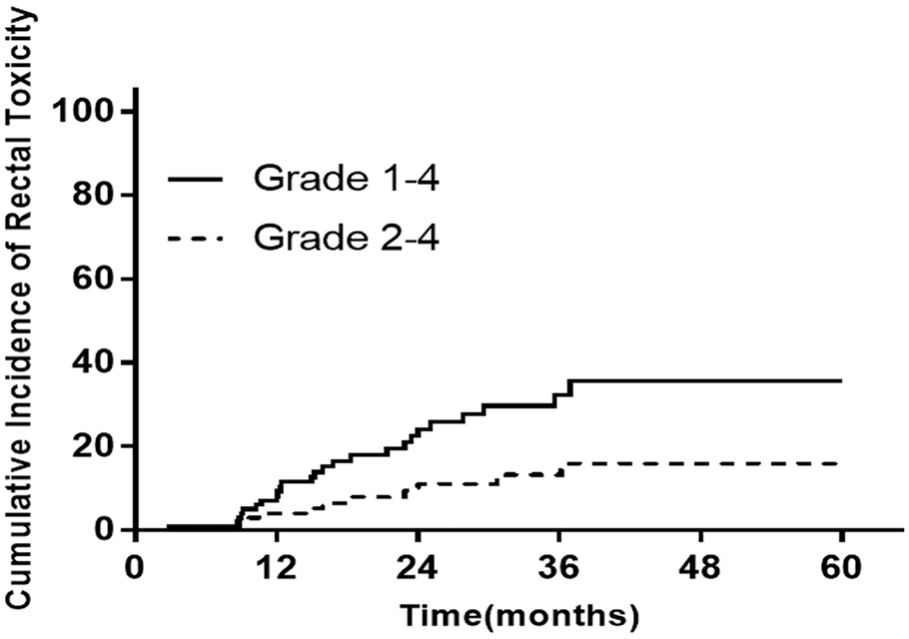
The incidence of late rectum morbidity in patients with locally advanced cervical cancer at 1/3/5 years.

The median follow-up time was 72.3 months. The incidence of late rectum morbidity in patients with locally advanced cervical cancer is presented in **Figure 1** and **Table 2**. The 1-, 3-, and 5-year incidence of rectum morbidity with grade 1–4 was 12.7, 30.0, and 31.8%, respectively. The 1-, 3-, and 5-year incidence of rectum morbidity with grade 2–4 was 8.2, 17.3, and 18.2%, respectively.

**Table 2:** The incidence of late rectum and bladder morbidity in patients with locally advanced cervical cancer at 1/3/5 years according to RTOG criteria.

RTOG	G0	G1	G2	G3	G4
1 year					
Rectum	96(87.3)	5(4.5)	3(2.7)	6(5.5)	0(0)
3 years					
Rectum	77(70.0)	14(12.7)	9(8.2)	9(8.2)	1(0.9)
5 years					
Rectum	75(68.2)	15(13.6)	10(9.1)	9(8.2)	1(0.9)

RTOG, the Radiation Therapy Oncology Group; G, grade.

### Dose of OARs for Predicting the Toxicity Morbidity

The Probit model was used to establish the prediction models between ${\text{D}}_{2\text{c}{\text{m}}^{3}},{\text{D}}_{1\text{c}{\text{m}}^{3}},{\text{D}}_{0.1\text{c}{\text{m}}^{3}}$, and D_ICRU_ for rectum and the 1-, 3-, and 5-year toxicity morbidities. Results clearly highlighted significant association of the ${\text{D}}_{0.1\text{c}{\text{m}}^{3}}$ of rectum with 1- or 3- or 5-year incidence of rectum morbidity with grade 1–4 or grade 2–4, and also revealed a significant relationship between ${\text{D}}_{0.1\text{c}{\text{m}}^{3}}$ of rectum and 1-year incidence of rectum morbidity with grade 1–4 or grade 2–4. The specific findings were as follows.

**Figure 2:**
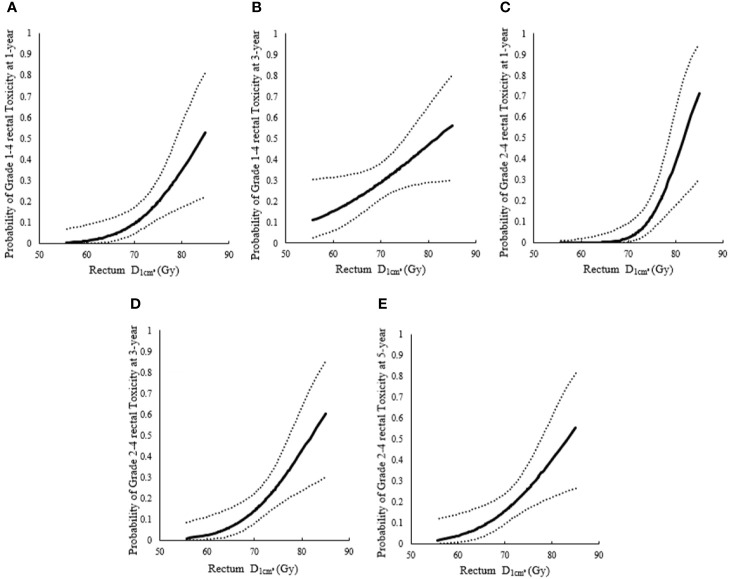
Probit models between incidence of rectum morbidity with different grade for rectum. **(A)** Probit models between 1-year incidence of rectum morbidity with grade 1–4 and ${\text{D}}_{1\text{c}{\text{m}}^{3}}$for rectum; **(B)** Probit models between 3-year incidence of rectum morbidity with grade 1–4 and ${\text{D}}_{1\text{c}{\text{m}}^{3}}$for rectum; **(C)** Probit models between 1-year incidence of rectum morbidity with grade 2–4 and ${\text{D}}_{1\text{c}{\text{m}}^{3}}$for rectum; **(D)** Probit models between 3-year incidence of rectum morbidity grade 2–4 and ${\text{D}}_{1\text{c}{\text{m}}^{3}}$for rectum; **(E)** Probit models between 5-year incidence of rectum morbidity with grade 2–4 and ${\text{D}}_{1\text{c}{\text{m}}^{3}}$for rectum.

**Figure 3:**
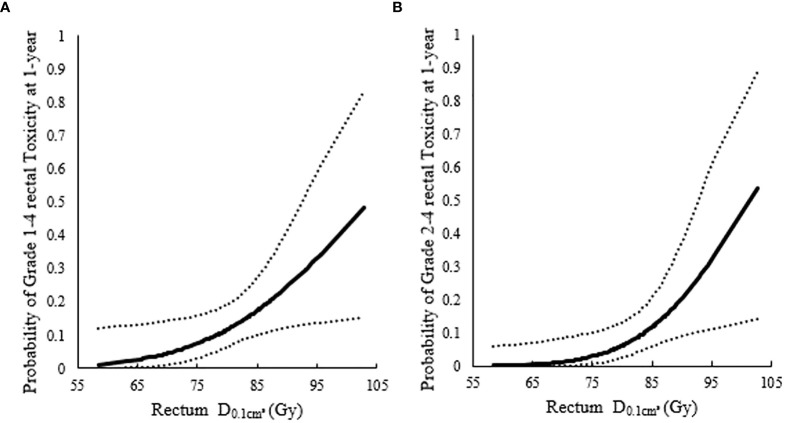
Probit models between 1-year incidence of rectum morbidity and highlighted significant association of the ${\text{D}}_{0.1\text{c}{\text{m}}^{3}}$ for rectum. **(A)** Probit models between 1-year incidence of rectum morbidity with grade 1–4 and highlighted significant association of the ${\text{D}}_{0.1\text{c}{\text{m}}^{3}}$ for rectum; **(B)** Probit models between 1-year incidence of rectum morbidity with grade 2–4 and highlighted significant association of the ${\text{D}}_{0.1\text{c}{\text{m}}^{3}}$ for rectum.

The 1-year incidence of rectum morbidity with grade 1–4 showed statistically significant relationship with ${\text{D}}_{1\text{c}{\text{m}}^{3}}\text{ and}{\text{D}}_{0.1\text{c}{\text{m}}^{3}}$ of rectum (see **Table 3** and **Figures 2A**, **3A**). According to the prediction models, ${\text{D}}_{1\text{c}{\text{m}}^{3}}$ was 70.29 (95%CI, 62.10–73.82) Gy_EQD2_ and ${\text{D}}_{0.1\text{c}{\text{m}}^{3}}$ was 78.25 (95%CI, 51.26–84.92) Gy_EQD2_ when the 1-year incidence of rectum morbidity with grade 1–4 was 10%. The 3-year incidence of rectum morbidity with grade 1–4 showed correlation with ${\text{D}}_{1\text{c}{\text{m}}^{3}}$ for rectum (see **Table 3** and **Figure 2B**). According to the prediction models, ${\text{D}}_{1\text{c}{\text{m}}^{3}}$ was 54.80 (95%CI, 28.62–63.23) Gy_EQD2_ when the 3-year incidence of rectum morbidity with grade 1–4 was 10%. The 1-year incidence of rectum morbidity with grade 2–4 showed correlation with ${\text{D}}_{1\text{c}{\text{m}}^{3}}\text{ and}{\text{D}}_{0.1\text{c}{\text{m}}^{3}}$ of rectum (see **Table 3** and **Figures 2C**, **3B**). According to the prediction models, ${\text{D}}_{1\text{c}{\text{m}}^{3}}$ was 74.18 (95%CI, 70.42–76.71) Gy_EQD2_ and ${\text{D}}_{0.1\text{c}{\text{m}}^{3}}$ was 83.70 (95%CI, 74.53–90.82) Gy_EQD2_ when the 1-year incidence of rectum morbidity with grade 2–4 was 10%. The 3-year incidence of rectum morbidity with grade 2–4 showed correlation with ${\text{D}}_{1\text{c}{\text{m}}^{3}}$ for rectum (see **Table 3** and **Figure 2D**). According to the prediction models, ${\text{D}}_{1\text{c}{\text{m}}^{3}}$ was 67.80 (95%CI, 58.12–71.11) Gy_EQD2_ when the 3-year incidence of rectum morbidity with grade 2–4 was 10%. The 5-year incidence of rectum morbidity with grade 2–4 showed correlation with ${\text{D}}_{1\text{c}{\text{m}}^{3}}$ of rectum (see **Table 3** and **Figure 2E**). According to the prediction models, ${\text{D}}_{1\text{c}{\text{m}}^{3}}$ was 66.37 (95%CI, 52.00–70.27) Gy_EQD2_ when the 5-year incidence of rectum morbidity with grade 2–4 was 10%.

**Table 3:** Probit model of relationships between ${\text{D}}_{2\text{c}{\text{m}}^{3}},{\text{D}}_{1\text{c}{\text{m}}^{3}},{\text{D}}_{0.1\text{c}{\text{m}}^{3}}$, D_ICRU_ for rectum and incidence of rectum morbidity grade 1–4, 2–4 at 1, 3, 5 years.

Parameter	Time	Grade	*β* [95%CI]	*Z*	*P*
$\text{Rectum }{\text{D}}_{2\text{c}{\text{m}}^{3}}$	1 year	G 1–4	−0.01 [−0.04, 0.04]	−0.17	0.865
		G 2–4	−0.01 [−0.06, 0.03]	−0.69	0.493
	3 year	G 1–4	0.01 [−0.03, 0.04]	0.41	0.680
		G 2–4	−0.01 [−0.05, 0.02]	−0.78	0.437
	5 year	G 1–4	0.01 [−0.03, 0.04]	0.21	0.835
		G 2–4	−0.02 [−0.05, 0.02]	−0.88	0.381
$\text{Rectum }{\text{D}}_{1\text{c}{\text{m}}^{3}}$	1 year	G 1–4	0.09 [0.03, 0.16]	2.83	0.005
		G 2–4	0.17 [0.07, 0.27]	3.24	0.001
	3 year	G 1–4	0.05 [0.01, 0.10]	2.09	0.037
		G 2–4	0.09 [0.03, 0.15]	3.07	0.002
	5 year	G 1–4	0.04 [−0.01, 0.08]	1.66	0.096
		G 2–4	0.08 [0.02, 0.13]	2.78	0.005
$\text{Rectum }{\text{D}}_{0.1\text{c}{\text{m}}^{3}}$	1 year	G 1–4	0.05 [0.01, 0.10]	2.19	0.029
		G 2–4	0.07 [0.01, 0.13]	2.42	0.015
	3 year	G 1–4	0.02 [−0.01, 0.05]	1.18	0.240
		G 2–4	0.03 [−0.01, 0.07]	1.44	0.150
	5 year	G 1–4	0.01 [−0.02, 0.05]	0.80	0.422
		G 2–4	0.02 [−0.02, 0.06]	1.13	0.260
Rectum D_ICRU_	1 year	G 1–4	−0.03 [−0.07, 0.02]	−1.17	0.241
		G 2–4	−0.03 [−0.09, 0.02]	−1.19	0.232
	3 year	G 1–4	0.01 [−0.03, 0.03]	0.03	0.973
		G 2–4	0.01 [−0.02, 0.04]	0.61	0.545
	5 year	G 1–4	0.01 [−0.02, 0.04]	0.41	0.683
		G 2–4	0.01 [−0.02, 0.05]	0.85	0.396

${\text{D}}_{2\text{c}{\text{m}}^{3}},{\text{D}}_{1\text{c}{\text{m}}^{3}},{\text{D}}_{0.1\text{c}{\text{m}}^{3}}$, *minimal dose to the maximally exposed 2 cm³, 1 cm³, 0.1 cm³ of organs at risk, respectively; D_ICRU_, dose delivered to the International Commission for Radiation Units and Measurements (ICRU) point, G, grade.*

### Evaluating the Predictive Value of Toxicity Morbidity for the Dose of OARs

The receiver operating characteristic (ROC) curve was used to evaluate and compare the predictive values of ${\text{D}}_{1\text{c}{\text{m}}^{3}}{\text{ and D}}_{0.1\text{c}{\text{m}}^{3}}$ of rectum for rectum morbidity at 1, 3, 5 years, see **Supplementary Figure S1**. **Table 4** showed the area under the curve (AUC) for the relationships between ${\text{D}}_{1\text{c}{\text{m}}^{3}},{\text{D}}_{0.1\text{c}{\text{m}}^{3}}$ of rectum and the incidence of rectum morbidity. In addition, comparison of AUC for the prediction of rectum ${\text{D}}_{1\text{c}{\text{m}}^{3}}$ and rectum ${\text{D}}_{0.1\text{c}{\text{m}}^{3}}$ to 1-year incidence of rectum morbidity with grade 1–4 showed no statistically significant difference (*P* = 0.338), (see **Table 4**). Comparison of AUC for prediction of rectum ${\text{D}}_{1\text{c}{\text{m}}^{3}}$ and rectum ${\text{D}}_{0.1\text{c}{\text{m}}^{3}}$ to the 1-year incidence of rectum morbidity with grade 2–4 showed no statistically significant difference (*P* = 0.083) (see **Table 5**).

**Table 4:** AUC of incidence of rectum morbidity grade 1–4.

	AUC [95%CI]	S.E.	*P*
1 year			
$\text{Rectum }{\text{D}}_{1\text{c}{\text{m}}^{3}}$	0.75 [0.61, 0.89]	0.07	0.002
$\text{Rectum }{\text{D}}_{0.1\text{c}{\text{m}}^{3}}$	0.71 [0.59, 0.84]	0.06	0.010
3 years			
$\text{Rectum }{\text{D}}_{1\text{c}{\text{m}}^{3}}$	0.61 [0.51, 0.73]	0.06	0.041

AUC, area under the curve; ${\text{D}}_{1\text{c}{\text{m}}^{3}}\text{ and}{\text{D}}_{0.1\text{c}{\text{m}}^{3}}$, *minimal dose to the maximally exposed 1 cm³, 0.1 cm³ of organs at risk, respectively.*

**Table 5:** AUC of incidence of rectum morbidity grade 2–4.

	AUC [95%CI]	S.E.	*P*
1 year			
$\text{Rectum }{\text{D}}_{1\text{c}{\text{m}}^{3}}$	0.88 [0.81, 0.96]	0.04	<0.001
$\text{Rectum }{\text{D}}_{0.1\text{c}{\text{m}}^{3}}$	0.80 [0.69, 0.91]	0.05	0.003
3 years			
$\text{Rectum }{\text{D}}_{1\text{c}{\text{m}}^{3}}$	0.74 [0.61, 0.86]	0.07	0.001
5 years			
$\text{Rectum }{\text{D}}_{1\text{c}{\text{m}}^{3}}$	0.08 [0.02, 0.13]	0.03	0.005

AUC, area under the curve; ${\text{D}}_{1\text{c}{\text{m}}^{3}}\text{ and}{\text{D}}_{0.1\text{c}{\text{m}}^{3}}$, *minimal dose to the maximally exposed 1 cm³, 0.1 cm³ of organs at risk, respectively.*

## Discussion

LRC is regarded as a major late side effect in patients with locally advanced cervical cancer who underwent treatment with EBRT followed by brachytherapy boost (10). The International Commission on Radiation Units and Measurements (ICRU) reference point for rectum has been used as the standard dose specific point. Several studies have shown a positive correlation between the X-ray based ICRU rectal point dose and the occurrence of late rectal morbidity (2, 3). However, there is an enormous variation in the dose distribution in the adjacent OARs walls when using definitive radiotherapy with brachytherapy, and then the minimum dose in most of the irradiated tissue volumes: 0.1 cm³, 1 cm³, and 2 cm³, namely ${\text{D}}_{0.1\text{c}{\text{m}}^{3}},{\text{D}}_{1\text{c}{\text{m}}^{3}},{\text{D}}_{2\text{c}{\text{m}}^{3}}$, were introduced for reporting the dose in the second GEC-ESTRO recommendations (11). Many researchers have reported that typical brachytherapy-related morbidities showed correlation with these small absolute volumes doses (12, 13). The dose–volume relationship for late rectal toxicity after ICBT has been systematically investigated before (14), but fewer studies have revealed the related outcomes of locally advanced cervical cancer treatment with IC/IS-BT. In the present study, the rectal D_ICRU_, ${\text{D}}_{0.1\text{c}{\text{m}}^{3}},{\text{D}}_{1\text{c}{\text{m}}^{3}}and{\text{D}}_{2\text{c}{\text{m}}^{3}}$ were calculated and the efficacy of these dose–volume histogram (DVH) parameters for predicting LRC in cervical carcinoma patients treated with definitive radiotherapy followed by IC/IS-BT were compared.

The prediction models between ${\text{D}}_{2\text{c}{\text{m}}^{3}},{\text{D}}_{1\text{c}{\text{m}}^{3}},{\text{D}}_{0.1\text{c}{\text{m}}^{3}}$, and D_ICRU_ for rectum and 1-year toxicity morbidity were established in this research. Consequently, positive dose–response relationships were observed between ${\text{D}}_{1\text{c}{\text{m}}^{3}}\;\mathrm{or}\;{\text{D}}_{0.1\text{c}{\text{m}}^{3}}$ and incidence of 1-year toxicity morbidity with grade 1–4, grade 2–4 LRC. Nevertheless, no positive dose–response relationships were observed between ${\text{D}}_{2\text{c}{\text{m}}^{3}}$, D_ICRU_ and with any grade incidence of LRC. This study also found that ${\text{D}}_{1\text{c}{\text{m}}^{3}}$ was predictive of a 3-year toxicity morbidity with grade 1–4 and grade 2–4 LRC, and 5-year toxicity morbidity with grade 2–4 LRC. Similarly, no positive dose–response relationships were observed between ${\text{D}}_{2\text{c}{\text{m}}^{3}}$, D_ICRU_ and with any grade incidence of LRC. Georg et al. (15) have found that the incidence of side effects of rectum was time-related; all rectal side effects have been developed within the first 3 years after treatment, and the majority of newly diagnosed side effects associated with rectum arise within the first 2 years. Thereafter, the incidence rates of LRC tended to stabilize during follow-up years 3–5. This meant that the incidence of rectal side effects was dynamically changed in short-term after treatment, while the DVH parameters remained constant. This might explain as to why different DVH parameters of rectum are considered as predictors for the incidence of rectal side effects during different time periods. It also concluded ${\text{D}}_{1\text{c}{\text{m}}^{3}}$ as a reliable indicator for predicting early and later grade 2–4 LRC.

Regarding the rectal D_ICRU_ hypothetical point determined by 2D image, it cannot directly represent the highest dose absorbed by the rectum (16). This explains that the resulting D_ICRU_ has very little for predicting the morbidity problem of rectum.

${\text{D}}_{2\text{c}{\text{m}}^{3}}$ showed a strong correlation for predicting the value of rectal ${\text{D}}_{2\text{c}{\text{m}}^{3}}$ dose with LRC (17, 18). Unfortunately, no correlation was observed in this research between ${\text{D}}_{2\text{c}{\text{m}}^{3}}$ and the incidence of 1-year with grade 1–4, grade 2–4 LRC, and even the incidence of 3/5year grade 1–4, grade 2–4 LRC. Nevertheless, the value of predicting late rectal toxicity for ${\text{D}}_{2\text{c}{\text{m}}^{3}}$ cannot be ignored or denied. The results obtained from several research studies (19) showed that ${\text{D}}_{2\text{c}{\text{m}}^{3}}$ was higher in patients with severe side effects. They also compared the severe side effects and mild adverse events by chi-square rather than Probit regression (in our study). But it became clear that high dose rectal ${\text{D}}_{2\text{c}{\text{m}}^{3}}$ could deteriorate LRC. In Mazeron et al. study (17), significant correlations were observed between rectal morbidity and ${\text{D}}_{2\text{c}{\text{m}}^{3}}$ and other DVH parameters by Probit model, which once again confirmed ${\text{D}}_{2\text{c}{\text{m}}^{3}}$ as an important predictive factor for LRC. Georg et al. (4) have demonstrated that the parameters ${\text{D}}_{2\text{c}{\text{m}}^{3}}\text{ and}{\text{D}}_{1\text{c}{\text{m}}^{3}}$ showed a good predictive value for rectal toxicity. Similarly, they enrolled 141 cervical cancer patients who received similar treatment regimen with us and evaluated the predictive value of rectal ${\text{D}}_{2\text{c}{\text{m}}^{3}},{\text{D}}_{1\text{c}{\text{m}}^{3}},{\text{D}}_{0.1\text{c}{\text{m}}^{3}}$, D_ICRU_ for LRC. In their research, significant differences of all DVH parameters and D_ICRU_ were observed between with and without LRC, but only ${\text{D}}_{2\text{c}{\text{m}}^{3}}\text{ and}{\text{D}}_{1\text{c}{\text{m}}^{3}}$ in major side effects were shown to be significantly higher than minor side effects. Furthermore, these studies also supported that high doses (>70–80 Gy) of ${\text{D}}_{0.1\text{c}{\text{m}}^{3}}$ might be associated with local effects such as ulceration, necrosis, and fistula, whereas intermediate doses (60–70 Gy), which was represented by ${\text{D}}_{2\text{c}{\text{m}}^{3}}$, would be associated with fibrosis, telangiectasia, or inflammation (20). It can be seen that both ${\text{D}}_{2\text{c}{\text{m}}^{3}}\text{ and}{\text{D}}_{1\text{c}{\text{m}}^{3}}$ are important indicators of LRC in locally advanced cervical cancer patients with IC/IS-BT. However, a clear association of ${\text{D}}_{2\text{c}{\text{m}}^{3}}$ with LRC was not found, and this might be due to limited number of patients and different scores in our work. Another reason for this is that the technique of IC/IS-BT might play an indispensable role. The EMBRACE (21) found a strong correlation between ${\text{D}}_{2\text{c}{\text{m}}^{3}}\text{ and}{\text{D}}_{0.1\text{c}{\text{m}}^{3}}$, but not for an individual at patient level, and the use of needles can cause a rectal wall hot spot and a major difference between ${\text{D}}_{2\text{c}{\text{m}}^{3}}\text{ and}{\text{D}}_{0.1\text{c}{\text{m}}^{3}}$. In our study, the patients enrolled underwent treatment with IC/IS-BT, which resulted ${\text{D}}_{0.1\text{c}{\text{m}}^{3}}$ as a better indicator that reflects the real hot spot distribution than the ${\text{D}}_{2\text{c}{\text{m}}^{3}}$ in patients who received IC/IS-BT.

Similarly, ${\text{D}}_{0.1\text{c}{\text{m}}^{3}}\text{, }and{\text{D}}_{1\text{c}{\text{m}}^{3}}$ were more sensitive for predicting the hot spot in the rectal wall. We observed that the ${\text{D}}_{1\text{c}{\text{m}}^{3}}$ was 74.18 (70.42–76.71) Gy_EQD2_, 67.80 (58.12–71.11) Gy_EQD2_, 66.37 (52.00–70.27) Gy_EQD2_ when the 1-, 3-, and 5-year incidence of rectal morbidity with grade 2–4 was 10% based on the Probit model in our research. Thus, the patients with rectum ${\text{D}}_{1\text{c}{\text{m}}^{3}}$>66.37–74.18 Gy_EQD2_ should be closely observed for grade 2–4 late rectal morbidity.

This study has the following limitations: firstly, this was a single-center, retrospective study with a small sample size, and a multi-center study should be conducted in the future. Furthermore, most of the advanced-stage diseases in the European and American countries are treated with a definitive chemoradiation, but in our country, selective cases with stage IIB are treated with neoadjuvant chemotherapy followed by radical hysterectomy according to the 2019 NCCN guidelines (22) and ESMO Clinical Practice Guidelines (23). Nearly half of the patients received neo-adjuvant chemo with a hope to render them resectable in our research. Therefore, patients, who were still inoperable after receiving neoadjuvant chemotherapy, were transferred to our department to receive definitive radio(chemo)therapy. Further studies are needed to clarify whether treatment with neoadjuvant chemotherapy might influence the target dose delivery, the prognosis, and patient survival. Thirdly, contouring could not be performed, and planning based on MRI at each treatment session, as nearly 30% of treatments in our study received CT guided brachytherapy. The accuracy of OARs delineated based on CT images was less than that of MRI images. The reasons for these are due to the result of uncertainty of dose accurate evaluation.

## Conclusion

${\text{D}}_{1\text{c}{\text{m}}^{3}}\text{ and}{\text{D}}_{0.1\text{c}{\text{m}}^{3}}$ were considered as useful dosimetric parameters for predicting the risk of grade 1–4 LRC at 1 year, and ${\text{D}}_{1\text{c}{\text{m}}^{3}}$ might be an indicator for predicting 3–5 years LRC with grade 2–4. A rectal ${\text{D}}_{1\text{c}{\text{m}}^{3}}$ above 66.4 Gy_EQD2_ may result in higher incidence of LRC > grade 2 using a Probit-type fitting.

## Data Availability Statement

The original contributions presented in the study are included in the article/**Supplementary Material**. Further inquiries can be directed to the corresponding author.

## Author Contributions

GC conceived of, designed, and supervised the study. NZ wrote the manuscript. YL, DH, and XG collected the data and analyzed the data. ZM and WY provided technical assistance with the study. All authors contributed to the article and approved the submitted version.

## Funding

This work was partially supported by grants from the National Natural Science Foundation of China [grant numbers 82073331, 81201737, 31600679,81703034]; Project of Science and Technology Department of Jilin Province (grant number 20190303151SF); and Horizontal Project of Jilin University [grant numbers 2019YX435, 2019155].

## Conflict of Interest

The authors declare that the research was conducted in the absence of any commercial or financial relationships that could be construed as a potential conflict of interest.

## Publisher’s Note

All claims expressed in this article are solely those of the authors and do not necessarily represent those of their affiliated organizations, or those of the publisher, the editors and the reviewers. Any product that may be evaluated in this article, or claim that may be made by its manufacturer, is not guaranteed or endorsed by the publisher.

## Supplementary Material

Supplementary Figure 1 ROC analysis for different significant features with incidence of rectum morbidity. **(A)** ROC analysis for D1 cm³, D0.1 cm³ for rectum with rectum morbidity grade 1–4 at 1, 3, 5 years. **(B)** ROC analysis for D1 cm³, D0.1 cm³ for rectum with rectum morbidity grade 2–4 at 1, 3, 5 years.
