# Supplementary figures and images for: The Relationship Between Late Morbidity and Dose–Volume Parameter of Rectum in Combined Intracavitary/Interstitial Cervix Cancer Brachytherapy: A Mono-Institutional Experience
Source: Front Oncol. 2021 Jul 23;11:693864. doi: 10.3389/fonc.2021.693864 (PMC8343064; doi:10.3389/fonc.2021.693864)

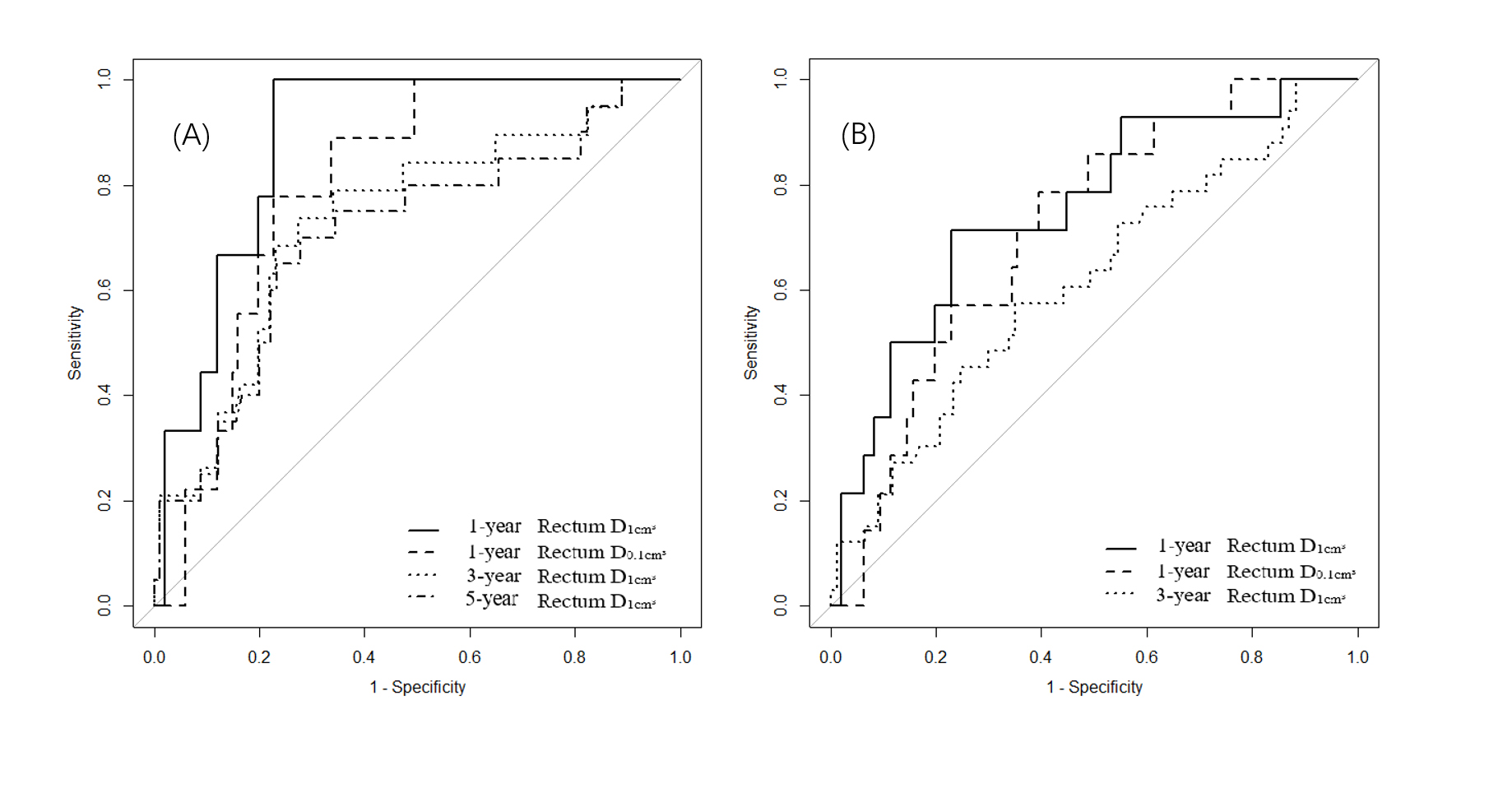

Supplement: Supplementary Figure 1 — ROC analysis for different significant features with incidence of rectum morbidity. (A) ROC analysis for D1 cm³, D0.1 cm³ for rectum with rectum morbidity grade 1–4 at 1, 3, 5 years. (B) ROC analysis for D1 cm³, D0.1 cm³ for rectum with rectum morbidity grade 2–4 at 1, 3, 5 years. [file Image_1.jpg]
