# Supplementary material for: The Relationship Between Late Morbidity and Dose–Volume Parameter of Rectum in Combined Intracavitary/Interstitial Cervix Cancer Brachytherapy: A Mono-Institutional Experience
Source: Front Oncol. 2021 Jul 23;11:693864. doi: 10.3389/fonc.2021.693864 (PMC8343064; doi:10.3389/fonc.2021.693864)
Supplement: Supplementary file 2 [file Table_1.docx]

**Supplementary Table.1** Patient and tumor characteristics and radiotherapy regimen

|  | | No. of patients | n/% |
| --- | --- | --- | --- |
| Histology | |  |  |
|  | Squamous cell Cancer | 104 | 94.5 |
|  | Others | 6 | 5.5 |
| FIGO stage | |  |  |
|  | Ⅰ B2 | 6 | 5.5 |
|  | Ⅱ A2 | 20 | 18.2 |
|  | Ⅱ B | 59 | 53.6 |
|  | Ⅲ A | 8 | 7.3 |
|  | Ⅲ B | 14 | 12.7 |
|  | Ⅳ A | 3 | 2.7 |
| External irradiation dose（Gy） | |  |  |
|  | 4320 | 1 | 0.9 |
|  | 4500 | 103 | 93.6 |
|  | 4680 | 1 | 0.9 |
|  | 5040 | 4 | 3.6 |
|  | 5580 | 1 | 0.9 |
| External irradiation technique | |  |  |
|  | IMRT | 88 | 80.0 |
|  | 3D-CRT | 22 | 20.0 |
| Number of brachytherapy | |  |  |
|  | 2 | 3 | 2.7 |
|  | 3 | 2 | 1.8 |
|  | 4 | 99 | 90.0 |
| Guide-image for brachytherapy | |  |  |
|  | CT | 144 | 32.9 |
|  | MRI | 294 | 67.1 |
| HR-CTV volume | |  |  |
|  | ≤30cm³ | 42 | 38.2 |
|  | ＞30cm³ | 68 | 61.8 |

FIGO= International Federation of Gynecology and Obstetrics; IMRT= Intensity-Modulated Radiotherapy; 3D-CRT= Three-Dimensional Conformal Radiotherapy; HR-CTV= High-Risk Clinical Target Volume.
